# Perspectives of HIV-positive and -negative people who use drugs regarding the criminalization of HIV non-disclosure in Canada: a qualitative study

**DOI:** 10.1186/s12889-020-09291-3

**Published:** 2020-08-10

**Authors:** Cara Ng, Koharu Loulou Chayama, Andrea Krüsi, Will Small, Rod Knight

**Affiliations:** 1 British Columbia Centre on Substance Use, 4th Floor, 1045 Howe Street, Vancouver, B.C V6Z 2A9 Canada; 2 Department of Medicine, University of British Columbia, Vancouver, Canada; 3 Centre for Gender and Sexual Health Equity, Vancouver, Canada; 4 Faculty of Health Sciences, Simon Fraser University, Burnaby, Canada

**Keywords:** People who use drugs, Criminalization, HIV, Non-disclosure, Stigma

## Abstract

**Background:**

While previous research has identified how criminalization of HIV non-disclosure can have deleterious effects on those living with HIV, the perspectives of people who use drugs – a population disproportionately affected by HIV– should be more meaningfully considered in these discussions.

**Methods:**

Using constant comparative techniques, data from 60 interviews with men and women living with and without HIV and who use drugs in Vancouver were analyzed to explore their perceptions about Canada’s HIV non-disclosure legal framework.

**Results:**

Participants’ perspectives on the framework involved three themes: understandings of HIV risk; HIV-related stigma; and their own experiences with HIV. While several participants favored the punitive character of the legal framework, these arguments were premised on misinformed and stigmatized assumptions regarding HIV.

**Conclusions:**

The paper concludes by discussing the challenges and opportunities for resisting HIV stigma and misconceptions about HIV within the context of personal accounts that, at times, support criminalization of non-disclosure.

## Background

Within and across many global settings, the non-disclosure of human immunodeficiency viruses (HIV) during sexual encounters remains a criminalized act. Specifically, 68 countries in the world, including Canada, criminalize the “non-disclosure, exposure or transmission” of HIV, while 33 other countries have applied different criminal provisions in similar instances [1]. In 2017, Canada ranked as having the third largest number of recorded prosecutions for alleged HIV non-disclosure globally [1–4]. Within the Canadian legal framework, HIV non-disclosure can result in charges of aggravated sexual assault or attempted murder. There have been approximately 184 people who have faced charges related to non-disclosure between 1989 and 2016 in this country, with the majority occurring since 2004 [5]. The legal framework for prosecuting HIV non-disclosure was first established in the precedent-setting *R v. Cuerrier 1998* decision in which the Supreme Court of Canada ruled that people living with HIV are legally required to disclose their HIV positive status in cases of “significant risk of serious bodily harm”, regardless of whether transmission had occurred [6].

In the early 2010s, the Supreme Court was revisiting the meaning of “significant risk”, with arguments suggesting this terminology can lead to ambiguity and therefore contribute to the legal framework being differentially and unevenly applied [7, 8]. Indeed, many criticisms have arisen in terms of how the legal framework is unfairly applied, including how charges tend to be concentrated among racialized people [9, 10] as well as how it is not uniformly applied across various levels of the judicial system (e.g., across different civil and/or provincial jurisdictions). In a 2012 decision during two cases of appeal (*R v. Mabior* and *R v. DC*), the Supreme Court ruled that individuals living with HIV must disclose their HIV positive status in sexual situations where “a realistic possibility of HIV transmission” occurred [11].

Unfortunately, from a medical perspective, the judicial application of this current legal framework ruling – a “realistic possibility” of transmission – does not sufficiently or consistently account for the current ‘intervention landscape’ of HIV treatment and prevention [1, 12–14]. It is now widely accepted that “undetectable viral loads are untransmittable” (i.e., U=U) [15] – a consensus that is firmly established, including through data published in 2016 from two large-scale clinical trials (PARTNERS and HPTN-052) [12, 13, 16]. Within some rulings under the “realistic possibility” framework, people who are HIV-positive can be protected from criminalization, but the burden of proof that a “realistic possibility” of transmission did not occur is high and rests entirely on the person living with HIV to demonstrate and prove that they met the standards of risk. Specifically, this framework has been interpreted by subsequent judicial rulings to require people living with HIV to be able to prove that they fulfilled two requirements during sexual encounters in which they did not disclose their HIV status in order to avoid being convicted: first, that they had low or suppressed viral loads at the time of the sexual encounter; and, second, that they also used a condom. As such, despite the reality that many people living with HIV have undetectable viral loads and transmission is no longer empirically possible when viral loads are low, under the current application of the legal framework, even those who have undetectable viral loads can be charged with serious criminal offences and be sentenced to significant time in prison if they cannot prove that they had an undetectable viral load and that they used a condom at the time of the encounter in which disclosure did not occur [14, 17, 18]. In late 2018, the federal government issued new prosecutorial guidelines to address the “over criminalization” of HIV non-disclosure however these guidelines are not binding outside the Yukon, Northwest Territories, and Nunavut [19].

### The Criminalization of HIV

While the legal framework around HIV non-disclosure is ostensibly deployed to ‘govern’ and prevent HIV transmission, human rights activists and people living with HIV have drawn attention to the ways this legal framework both fails to prevent HIV transmission and increases overall harm – and, that harms are unequally experienced by socially vulnerable and disadvantaged populations. For example, as is the case with the criminalization of drug use, research has pointed to the ways the criminalization of non-disclosure leads to the uneven application of criminal charges among Indigenous and Black people, as well as among LGBTQ and poor communities living with HIV [10, 20, 21].

Previous research has also identified how laws and policies that criminalize HIV can serve as structural drivers of HIV stigmatization [14, 22]. These include, for example, studies that have identified how Canada’s legal framework may negatively impact trust and confidentiality within patient-clinician encounters, as well as how it can reduce access to regular testing for those who do not know their status and affect retention within the HIV cascade of care for those living with HIV [22, 23]. There have been studies that have focused attention on the ways legal frameworks increase overall harm for those living with HIV (e.g., stigma, prejudice) while doing little to prevent HIV transmission [24, 25]. Others have also argued that instead of focusing on the shared responsibility that those living without HIV – including communities and society more broadly – have in terms of preventing HIV transmission, the criminalization of non-disclosure emphasizes individualized notions of responsibility of HIV prevention. This leaves the onus of HIV prevention left exclusively upon those living with HIV [21, 26], thereby contradicting decades of public health HIV prevention messaging.

### Everyday understandings about HIV non-disclosure

Amidst concerns about the various effects of the criminalization of HIV non-disclosure, including the unintended effects of stigma and other corresponding negative health and social outcomes, in the Canadian context, surprisingly little is known about how the legal framework is actually understood, perceived and experienced by individuals in a society ‘governed’ by these frameworks. There have been a few important studies regarding the ways in which the criminalization of non-disclosure impacts upon marginalized groups in Canada. These studies have focused on the ways people living with HIV from a range of social locations (e.g., people who use drugs, refugee and immigrant women, cis and trans women) understand non-disclosure legal frameworks and negotiate it within the context of their own lives and sexual relationships [27–29]. In a recent article, Knight et al. [14] explored the impacts of HIV criminalization legal frameworks on the perspectives of and experiences with HIV risk among 85 HIV-negative men and/or men who were unaware of their serostatus. In that study, the authors identified two narratives, including narratives of “justification” and “interrogation” in relation to existing legal frameworks around HIV criminalization. Within the *interrogation* narrative, the authors identified how participants tended to problematize the acceptability of criminalizing HIV non-disclosure because they felt that the framework created unjust barriers to HIV testing uptake (for those unaware of their status) and HIV treatment and care (for those living with HIV). Within the *justification* narrative, the authors noted that HIV literacy was often very low, including references to HIV as a “death sentence”. Those who advanced the *justification* narrative also tended to suggest that the legal framework provides both punishment and deterrence, which were perceived to supersede considerations about barriers to care for both HIV-positive and -negative individuals.

While examples of research in this area clearly exist, authors of this article are not aware of any qualitative studies regarding the perspectives of both people living with and without HIV who use illicit drugs on the non-disclosure legal framework in the Canadian setting. Furthermore, people who inject drugs have poor access to HIV prevention, treatment, and care due to the continued criminalization of drug use and ongoing stigma against people who use drugs [30, 31]. Of all new HIV diagnoses in BC in 2016, 7.1% occurred among people who inject drugs (PWID) – despite only comprising approximately 1% of the population [32]. Given that this population encounters a variety of social and structural barriers to accessing health care, and are also vulnerable to contracting HIV due to repressive anti-drug policies that enhance HIV susceptibility and present barriers to care [33–36], understanding these perspectives is critical to closing health inequities.

While previous research has been helpful in identifying the impacts that HIV non-disclosure legal frameworks have on people living with HIV (e.g. increased violence and stigma), more information about how these frameworks influence the experiences and perspectives of both people living with and without HIV in the Canadian context are needed. This knowledge gap remains salient for people who use drugs. Therefore, the objective of this study was to examine the perspectives of people who use drugs about the HIV non-disclosure legal framework. To do so, the insights of 30 men and 30 women living with and without HIV who use drugs living in Vancouver, Canada were explored.

## Methods

### Study setting

Data collection activities were based in Metro Vancouver in the province of British Columbia (BC), Canada. The region’s total population in 2016 was 2,463,431 [37]. In line with historical trends in BC, men continue to have higher rates of HIV transmission than women. While the BC Centre for Disease Control reports that 7.1% of all new HIV diagnoses in BC occurred among PWID, this population has also recently experienced declining rates of the disease (e.g., 66 cases in 2008 compared to 16 cases in 2016), likely reflecting the success of recent expansion and sustained prevention efforts with this population [32]. For example, a “seek and treat” pilot program was launched in Vancouver and Northern BC in 2010, in order to expand access to HIV testing and treatment for BC residents [38]. Among PWID, 70.6% of new diagnoses were among men, and the greatest number of new infections occurred among those between 30 and 59 years of age [39] (94.1%). In BC, Indigenous people are also disproportionately affected by HIV; in 2015, they comprised 7.9% of new diagnoses in the province, while representing only 5% of the total population. Indigenous men comprised 4.5% of new diagnoses in the province among men, while Indigenous women comprised 27% of new diagnoses in the province among women [39]. Further, Indigenous populations in BC comprised 13% of opioid-related deaths in the province, despite making up only 3.4% of the population [40].

### Recruitment and data collection

Participants were recruited between February and September of 2017. HIV-negative PWID were recruited through the Vancouver Injection Drug Users Study (VIDUS) and HIV-positive PWID were recruited through the AIDS Care Cohort to Evaluate access to Survival Services (ACCESS) study. These cohorts require participants to be 18 years of age or older, provide written informed consent, and have used illicit (ACCESS) or injection (VIDUS) drugs prior to their enrolment [3, 4]. ACCESS and VIDUS participants have been recruited through community outreach, and both studies maintain follow-up rates of ≥85%. At baseline and semi-annual follow-up visits, participants complete questionnaires administered by a trained interviewer or nurse, and provide a blood sample for HIV and Hepatitis C virus (HCV) testing (VIDUS) or ongoing clinical monitoring of HIV disease progression (ACCESS).

Stratified purposeful sampling [41] was used to deliberately select participants from a variety of backgrounds and ‘profiles’ (e.g., by gender identity, ethno-cultural identity) and to identify those who may experience various vulnerabilities (e.g., previous or current injection drug use) with regards to HIV and HIV non-disclosure legal frameworks [42, 43]. It is important to note that the sampling distribution in terms of ethno-cultural and racial identity does not match the distribution of the general population in BC. For instance, while Indigenous people comprise 5.9% of the population in BC, in the study sample, they represent over half of participants (~ 51.67%) [37]. Their ‘over-representation’ in the sample likely reflects the co-existing issues of inequality (e.g., poverty, racism, colonization) faced by Indigenous communities in BC and Canada.

ACCESS and VIDUS frontline research staff approached individuals who were eligible and met the sampling framework to participate in qualitative interviews during their semi-annual or other follow-up visits to the field office. Eligibility criteria included being participants in VIDUS or ACCESS. Participants were flagged in the cohort database so that when they arrived at the field office for their visits, they would be approached by the front desk person and asked if they were interested in participating in the study. Research and nursing staff also determined eligibility by tracking and ‘flagging’ potential interviewees on the main or nursing questionnaire (i.e., if participants were found to meet the eligibility requirements through the questions posed in these questionnaires, they would be approached to do an interview). Participants were told that there was a study about the experiences of accessing HIV programs and services for people using drugs. At this point, prospective participants who expressed interest in participating in the study were referred to a member of the team who provided them with further information about the interview, confirmed their eligibility, and invited those who were eligible to participate in the study subsequent to providing written informed consent. Participants received a CAD$30 honorarium to compensate them for their participation. Ethics approval was obtained from the University of British Columbia’s Behavioural Research Ethics Board (#H16–00325).

Interviews were conducted within the research offices in Vancouver’s Downtown Eastside. Interviews lasted 30 to 60 min in duration and were audio-recorded. During each interview, participants were asked to share what they knew about the legal framework surrounding HIV non-disclosure in Canada. All participants were provided with some additional background information by the interviewer based on a script provided in the interview guide. Participants were also asked the opportunity to ask questions of clarification regarding Canada’s HIV non-disclosure legal framework. They were then invited to discuss how their understandings of HIV and HIV risk might relate to the broader context of Canada’s current legal framework pertaining to HIV non-disclosure. It is important to point out that while the interviews may have provided an educational component for the participants about the HIV non-disclosure legal framework for those who did not already know about it, this was not the intention. In order to remain as neutral as possible, the interview guide provided a script that was factual in nature regarding the HIV non-disclosure legal framework. All of the interviewers were experienced in interviewing and used open-ended questions while avoiding leading questions. See Table 1 for the questions and probes interviewers used to guide the discussion during interviews.

**Table 1:** Questions and probes on the topic of the criminalization of HIV non-disclosure

***PERSPECTIVES ON HIV CRIMINALIZATION***	
*In Canada, we have a law that criminalizes HIV+ individuals who do not disclose their HIV + status to sexual partners unless they use a condom AND have a ‘low’ viral load.* **During this section, I am not asking about what you have done in the past, but am interested in understanding your** ***opinions*** **on this issue.** Have you come across any information related to this law?	▪ Were you aware of this law? ▪ What do you think about this law? ▪ Where did you hear this information? ▪ Can you tell me about any accounts of HIV non-disclosure that might stand out in your memory?
In Canada HIV non-disclosure has most often been prosecuted as aggravated sexual assault. Aggravated sexual assault carries a sentence of jail time up to a maximum of life imprisonment and registration on the Sexual Offender Registry. This is one of the most serious crimes in the Canadian Criminal Code. In Canada, over 150 people have been prosecuted for HIV non-disclosure to date, even where no transmission has taken place and where in many cases the risk of transmission was considered to be very small. What are your initial thoughts on how HIV is treated in Canada in these sorts of circumstances?	▪ Tell me about your overall thoughts about this law. ▪ How do you think this law influences people living with HIV? ▪ Do you think that this law is unfair for certain groups of people? ▪ Do you think everyone is able to disclose their HIV status when they are going to have sex?

### Analysis

Audio recordings of the interviews were transcribed and accuracy checked by a third party and subsequently uploaded to NVivo12. This research is framed within a modified grounded theory methodology [44]. Co-authors (of whom one, RK, was involved in the interviewing process) read and re- read transcripts, employing constant comparative techniques [45, 46] to inductively derive themes relevant to the study. Constant comparative techniques were appropriate for this study since it was important for the themes to be directly constituted and informed by the patterns and contradictions that emerged from the qualitative data in the interviews. Specifically, the individual transcripts were compared and contrasted to identify patterns, and shared and divergent understandings were identified. Next, an open-coding approach was used in which coding was first organized into ‘trees’ to group the codes thematically. The emergent thematic codes focused on capturing the micro- (e.g., previous experiences), meso- (e.g., interpersonal relations) and macro (e.g., socio-cultural) factors [47] that shape participants’ opinions and behaviours related to the criminalization of HIV non-disclosure, with a specific focus on how understandings of HIV risk influence each participant’s perspectives regarding the legal framework.

Consistency of coding (inter-coder reliability) was assessed by team members and any discrepancies were discussed and resolved at research team meetings. Discrepancies between codes and coders were resolved through discussion and raw data was re-visited as needed during data meetings. Analytic memos were kept throughout the coding to document the analysis process as it developed and key decisions, and these were discussed throughout the analysis of the data, writing of this article, and drafting of this report. “Verification” strategies were also used to establish rigor in the approach to validate decisions about the analysis, including the emphasis of responsiveness and openness of all authors to the data in adjusting or revising the codes and presentation of each thematic to result in a consensus-driven representation of the findings.

The data analysis was guided by two overarching analytical questions, which were arrived at during the final stages of organizing data and data analysis: 1) How are participants’ perspectives on the criminalization of HIV non-disclosure shaped by understandings of societal stigma? 2) How are perspectives about the criminalization of HIV non-disclosure shaped by understandings of HIV and HIV risk in an era where “Undetectable = Untransmittable”? As this process unfolded, a final set of codes to identify the broad themes that emerged across the data set was established. In doing so, a thematic analysis was conducted with both an inductive analytic approach to develop initial coding schema and general themes, as well as deductive approaches in which findings were used to compare and contrast the existing literature pertaining to the perspectives and experiences of the non-disclosure legal frameworks [48, 49].

### Findings

In total, 60 participants (30 men; 30 women), who ranged in age from 33 to 73, were recruited to participate in in-depth, semi-structured interviews. Table 2 provides further information on the socio-demographic characteristics of the sample.

**Table 2:** Socio-demographic characteristics of study participants

	(n)	(%)
**Living with HIV**		
Yes	30	50%
No	30	50%
**Ethnicity**		
Indigenous/First Nations/Metis	31	~ 51.67%
White	25	~ 41.67%
Black/Caribbean/African	3	5%
Unspecified	1	~ 1.67%
**Gender identities**		
Woman	30	50%
Man	30	50%
**Age**		
30–39	7	~ 11.67%
40–49	24	40%
50–59	21	35%
60–69	5	~ 8.33%
70+	2	~ 3.33%
Unspecified	1	~ 1.67%
**Sexual Orientation**		
Gay	4	~ 6.67%
Straight	46	~ 77%
Two-Spirit	2	~ 3.33%
Bisexual	3	5%
Other	2	~ 3.33%
Unspecified	3	5%

“gender identities” categories are inclusive of both cisgender and transgender identities

### Overview of findings

During the interviews, participants’ perspectives were solicited on the legal framework that criminalizes HIV non-disclosure in Canada. During these discussions, some of the participants were more familiar than others with the specifics of the Canadian legal framework. However, almost all of the participants were aware that there could be legal implications for people living with HIV who do not disclose their status during sexual encounters. Below, findings in light of three interrelated themes are presented that emerged from the analysis of how participants discussed their perceptions of and experiences with the current legal framework as follows: (1) understandings of HIV risk; (2) stigmatized beliefs about people living with HIV; and (3) personal experiences with HIV and HIV risk. Each quotation is preceded by a short description of each participant’s socio-demographic profile and followed by a researcher-assigned numeric code.

1. *‘They have no right to play God with your life’: Understandings of HIV risk and an evolving HIV intervention ‘landscape’*

Participants were asked about how the risk of HIV transmission features within how they think about Canada’s HIV non-disclosure legal framework. As interviewers initiated these discussions, many of the HIV-negative participants told interviewers that they believed an HIV diagnosis would result in either the rapid decline of health or even death (e.g., progression to Acquired immunodeficiency syndrome, i.e., AIDS) and was therefore, in their view, analogous to physical assault or murder. For example, two participants explained:

*I mean, you can die from that [HIV]. And if the other person doesn’t know about it, then it’s like a murder charge kind of thing, you know what I mean?* (HIV-Negative, Woman, White; 0030)

*They didn’t ask you to give them that disease to them. I think they should all be thrown in jail.* (HIV-Negative, Woman, Indigenous/First Nations; 0028)

As interview discussion about HIV risk continued, participants described how they viewed the *act* of a person living with HIV not disclosing their status as featuring morally questionable behaviour – though the act of not disclosing was invariably underscored by concerns about risk of transmission, regardless of whether or not one is undetectable. For example, when asked if participants felt the legal frameworks should still apply to situations in which individuals had “low to no risk” of transmission, participants’ responses tended to emphasize that concerns about risk always needed to feature within their assessments of the legal frameworks:

*If they’re undetectable and they still have the virus, it can still be a risk factor. Yeah, but still they put the other person at risk without disclosing. They didn’t care whether they got it or not. They’re only thinking about their own sexual needs.* (HIV-Negative, Man, Indigenous/First Nations; 0012)

*I don’t think there’s very much difference. It’s still … it’s still irresponsible for the person who’s got HIV, you know … I know if I had it, I would be responsible and tell them, you know. Anybody that’s around me, like regardless of … what people think of me, it’s just the responsible thing to do, right? Because … it’s not something that I would want to catch.* (HIV-Negative, Man, Indigenous/First Nations; 0023)

***Respondent:*** *You should be going down for murder, I feel. You want to fucking fuck with someone’s life, then you’ve just murdered them. [ … .]*

***Interviewer:*** *And you can’t even get it in cases where – you know how we talked about like undetectable?*

***Respondent:*** *Yeah.*

***Interviewer:*** *So even if it’s really low to no risk?*

***Respondent:*** *Well, still, though. You didn’t tell them! They have no right to play God with your life.* (HIV-Negative, Woman, Indigenous/First Nations; 0011)

During these discussions, interviewers were frequently struck with how participants – particularly those not living with HIV – tended to express support for HIV non-disclosure legal frameworks based on misguided understandings of HIV ‘risk’ in the context of highly effective and available treatment in British Columbia.

Among those living with HIV, a very different understanding of HIV risk tended to emerge. For example, most participants living with HIV described that they were aware that when viral loads are undetectable, transmission is not possible. Nevertheless, some of those living with HIV continued to emphasize that they felt disclosing their HIV status to sex partners was something that one ought to do, regardless of the risks involved. These participants described that they wanted to disclose to partners so they could align more closely with virtues they valued, including honesty and compassion – something they described as being particularly important when a current or prospective romantic and/or sex partner was involved.

Among some participants living with HIV, however, significant misconceptions about HIV risk continued to emerge in the interviews, particularly during discussions about how one can be charged and sentenced to long terms in prison for not disclosing, including in situations in which an individual has a suppressed viral load but did not use a condom:

*Well, I think it’s imperative that people know* [even in cases where transmission is low]*, because you know, whether you’re going to get lucky that night, it’s their livelihood [health]. So, I mean, you change their lives if you … and you should respect that, because you know what happened to you, yourself, right?* (HIV-Positive, Man, White; 0033)

*They should get a lot more than that.... you’re giving somebody life, ruin somebody’s life.* (HIV-Positive, Woman, White; 0049).

*Well, I think it’s* [being charged with aggravated sexual assault for not disclosing when undetectable] *a good idea, because you’re basically playing with somebody else’s life. Because it’s a life. You have this disease for life, you know.* (HIV-Positive, Woman, Indigenous/First Nations; 0045)

Participants’ perspectives about HIV risk and the legal frameworks tended to largely stem from a ‘pre-Antiretroviral’ era that disavowed the current realities of the intervention ‘landscape’. As such, these perspectives were frequently underpinned by a misguided premise that there is always risk for transmission, including when individuals with HIV have low viral loads (less than 200 copies per milliliter of blood), which is not consistent with current understandings.

2) *‘They’re too ashamed of telling anybody’: Stigma and HIV non-disclosure*

At times, including when participants referred to how one’s life would be “ruined” or “played with” for those who acquire HIV, interviewers were left wondering how participants’ concerns relate to the health and/or social consequences of HIV acquisition. To better understand these perspectives, participants were asked about how HIV disclosures occur in “real world” settings. Almost all of the participants described how one’s HIV-positive status could make it too difficult to disclose within sexual encounters, and this was invariably based on the social implications (e.g., rejection) one may experience as a result of disclosing. For example, participants frequently described how the fear of sexual or social rejection could present real-world barriers for people living with HIV from being able to disclose their status to sex partners:

*I think with HIV, when you factor in that social stigma, like why should someone have to say like, ‘Oh, I’m HIV-positive,’ on the chance that person’s going to react really negatively or harshly?”* (HIV-Negative, Woman, White; 0021)

*I don’t think a lot of them do [disclose] because they’re afraid, you know. Like they’re afraid that they might lose their partners or whatnot.* (HIV-Positive, Man, Indigenous/First Nations; 0043)

As such, a subset of participants began to interrogate how one’s ‘decision’ not to disclose their HIV-positive status was deeply linked to features of social context that stigmatize HIV. Within these discussions, interviewers were often struck with how calls for ‘personal responsibility’ and the need to disclose ran counter to participants’ understandings and concerns about the profound stigma that actively limits – or, in many cases, *prevents* – the ability to disclose. For example, one participant described how, despite emphasizing that feelings of shame and fears of social repercussions represent critical barriers to disclosure, she felt those living with HIV should nevertheless be ‘responsible’ and disclose their status:

*One of the ways that it’s* [HIV] *contracted a lot, because they’re* [people living with HIV] *too ashamed of telling anybody that they’re positive and not being responsible enough to say, “Hey, I’m HIV positive. Maybe we should … take some responsible steps”, you know.* (HIV-Negative, Woman, White; 005)

Despite the widespread acknowledgement among participants that stigma limited how disclosure can occur, it became clear that many of the participants felt that negotiations about safer sex were, in ‘practice’, almost entirely the responsibility of individuals living with HIV. Within these discussions, some participants emphasized that concerns about stigmatization needed to be reconciled alongside the values they placed both on the autonomy and privacy of people living with HIV and the value they placed on protecting the health of ‘self’ and ‘others’. Nevertheless, as these discussions unfolded, participants tended not to describe how those living without HIV may also have responsibilities around safer sex practices, instead often returning to the need to compel those living with HIV to disclose their HIV status via mandatory and coercive enforcement mechanisms, i.e., legal frameworks. Here, participants tended to suggest that concerns about autonomy or privacy for people living with HIV – concerns all participants emphasized needed to be taken seriously – should be overruled in order to protect the health and well-being of those not living with HIV. One participant not living with HIV described the complexities involved in navigating between personal autonomy and the health and wellbeing of the broader community:

*I feel like the ability to choose when* [someone is going] *to disclose is important, and stigmatized facts about themselves and medical history is very important to protect. But also, that those kinds of considerations of autonomy and privacy stop being personal when they start affecting other people. And it’s just when and where it switches from the one to the other.* (HIV-Negative, Woman, White; 0021)

Some of the participants living with HIV described how the stigma associated with HIV reinforced how they interacted with sex partners, with many describing how the fear of both being criminally charged and socially and/or romantically rejected shaped situations that made them feel unsafe. One participant living with HIV described how past experiences of rejection from romantic partners resulted in him deciding to disclose his HIV status at the beginning of a romantic relationship in order to prevent future feelings of heartbreak should he become attached and then rejected again:

*When you like someone that much you’re gonna say “Oh, oh my God, I’ll lose them if I say I’m positive, I’ll probably lose them, right.” But if you tell them in the beginning at least it hurt less cause you’re not attached with the person yet.* (HIV-Positive, Man, White; 0048)

While all of the participants in the study felt HIV stigma represented a key barrier to those who are tasked with disclosing their HIV-positive status, two participants living with HIV argued that HIV stigma ‘no longer mattered’ to them, partially due to the evolving social context surrounding HIV. For example, two participants described:

*I’m pretty confident in who I am, right? So it doesn’t bother me what people think, so I tell people that I have HIV like pretty well right away... And the lifestyle I live, HIV is … it’s so common right? It’s got no stigma attached to it at all, right? You know?* (HIV-Positive, Man, White; 0034)

*Well, you know, before it* [HIV stigma] *might have been more of a problem, right? But nowadays, I don’t think it is, you know, because the education is more there. People were ashamed back then, even when I first got it. But now it’s, you know, it’s so open, you know. Like the HIV is so open now, you know. It’s not a shameful thing.* (HIV-Positive, Woman, Indigenous/First Nations; 0045)

Despite emphasizing that the negative effects of HIV stigma were from a previous era, these participants nevertheless expressed support for the legal frameworks that criminalize HIV non-disclosure. For these participants, a dissonance began to emerge in which the ‘progress’ made with regards to HIV treatment was undermined by the extent to which they continued to view HIV as an ‘exceptional’ infectious disease that, as a result, required a legal framework to ‘govern’ HIV risk and transmission. These discussions were often highly personal and revealed important histories about how those living with HIV connected their previous experiences with HIV and HIV risk to their understandings of the HIV non-disclosure legal frameworks. As such, as the interviews progressed, the stories of those living with HIV continued to emerge and inform their perceptions about the HIV non-disclosure legal framework.

3) *‘That’s what happened to me’: Personal experiences with HIV and HIV risk*

A number of participants expressed varying degrees of support for the criminalization of HIV non-disclosure based both on their own personal experiences with HIV, as well as based on stories about HIV non-disclosure circulating among their peers and via the media. As participants provided examples that they had heard about, they tended not to know the individuals involved in the stories directly. Nevertheless, participants described how these situations featured purported acts of non-disclosure that were committed with the clear intention of a person living with HIV to purposefully harm, i.e., infect, others by exposing them to HIV:

*I heard of a couple of other girls down here that were going out and having sex with men, and they knew they had HIV and they intentionally... I think they’re still out there somewhere. And were spreading it, and they should be put down [euthanized] … That’s a serial killer. [Mm-hmm] a serial killer. And yeah, those people should be extinguished.* (HIV-Negative, Man, Indigenous/First Nations; 0025)

*You know, there was one guy that went around … . I don’t know if it was in the States or in Canada, but – I think it was in Canada, but he exposed, what, 30 girls, or so? And oh, man, it was crazy. Fuck, he just went out, and he knew he had it. And they were after him, a manhunt on him. And they finally got him.* (HIV-Negative, Man, White; 0026)

As such, participants tended to buttress a ‘pro-criminalization’ stance on HIV non-disclosure based on somewhat sensationalized accounts and stories. For example, as participants reflected on the stories they had heard about, some tended to construct narratives that featured people living with HIV as inherently ‘bad’ and ‘to be feared’ because they could easily and without warning choose to purposefully transmit the virus. While this concern tended to be put forth by those living without HIV, several of those living with HIV reflected on how these perceptions of the reprehensible ‘other’ ran up against their own experiences and observations of others living with HIV:

*I think the law should be you should have to be open no matter what. [ … ] If somebody wants to give you HIV they can give you HIV and you’re not gonna do nothing about it. They want to stab you with a rig full of their blood what can you do? It’s a pretty easy way to transmit it. But, like in social living as opposed to all these other transmittable diseases like other STDs right, it of course is a very low risk with HIV now. But people actually people know that now and they’re being high risk because of that and that worries me too.* (HIV-Positive, Man, White; 0056)

Many of the participants reflected on how they felt they likely acquired HIV from a partner who did not disclose their positive status, though all of the participants reported that they were unsure and unaware of the specific encounter that may have resulted in their own acquisition of HIV – experiences that occurred up to 20 years previously. A sub-set of the participants living with HIV described how they would have liked their previous sexual partners to have disclosed their HIV-positive status because they could have either declined to have sex or used precautions (e.g., condoms). For example, one participant reflected on how her own experience acquiring HIV features within how she thinks about HIV risk and the application of the legal framework surrounding HIV non-disclosure:

*Where no transmission takes place, it’s kind of a little harsh. But it depends on the situation. They still … whether transmission took place or not, you still didn’t tell them. You took their choice away … . And that’s not right in my mind. It’s really not right. Like, if I’m ever intimate with anybody, I tell them right away. I’ve got HIV. Now you deal with it. It’s your choice. It’s in your court. Because, like I said, I was perturbed when nobody told me.* (HIV-Positive, Woman, undisclosed ethnicity; 0038)

As the interviews came to an end, some participants living with HIV pointed out a conundrum or dilemma of sorts, which functions as a catch-22, that they had identified based on their own experiences living with HIV. Specifically, these participants identified how the legal framework in Canada is implicated in increasing HIV stigmatization broadly and, as a result and at the same time, (re) produces a set of social conditions that makes disclosure extremely difficult and unsafe (e.g., due to fears of violence). As such, several participants pointed out how they felt the law itself is inherently paradoxical and counterproductive, particularly within the pursuit of “governing” HIV disclosure. For example, one participant living with HIV described how the criminalization of HIV non-disclosure is, in itself, a stigmatizing act. Here, the participant described how the law seems to be premised on concerns that those living with HIV would purposefully transmit concurrently frames people living with HIV as being inherently ‘bad’ people:

*I think* [the legal framework] *makes people with HIV look really bad. Like, you know, it makes them look like they don’t give a shit about nothing. Like I wouldn’t want to hurt somebody. I don’t know why people would want to hurt somebody. I don’t even know how people do it. I mean, like if I would have known that, that person could have got charged, you know, like that I slept with that time. Like I mean, I probably would have like. I probably would have said, “No, don’t charge him.” I mean, that would be crazy.* (HIV-Positive, Man, Indigenous/First Nations; 0044)

## Discussion

The findings reveal several important insights with regards to PWUD’s perspectives and experiences regarding legal frameworks surrounding HIV non-disclosure in Canada. First, participants tended to characterize the benefits of the legal framework with regards to misinformed understandings of HIV risk, as well as based on stigmatized beliefs about people living with HIV. As such, participants who described favoring the legal framework tended to premise their rationale based on misinformed and stigmatized assumptions about HIV and HIV risk. Others described how their own personal experiences with HIV and HIV non-disclosure informed how they thought about the legal framework, including how they either justified or interrogated the legal framework.

Many of the participants in the study described how they felt the legal frameworks could serve an important role in compelling those living with HIV to disclose their status with sexual partners. Within these examples, several participants described how they felt the interests of those living without HIV may need to ‘supersede’ the interests of those living with HIV (e.g., privacy, safety). While a few participants living with HIV understood that “Undetectable = Untransmittable”, they nevertheless tended to express support about the need to disclose one’s HIV status, frequently suggesting that the legal frameworks should be founded on the need to uphold and govern a set of social conditions in which those living with HIV could do no harm to others. As the interviews progressed, interviewers were frequently struck with how concerns about ‘doing no harm’ ran parallel to the very reasons and justifications participants living with HIV used in their conscientious efforts to avoid transmitting the virus.

While relational values are important to consider, in light of a criminalization legal framework that presumes that the sexual behaviours of people living with HIV need “governing”, it is important to further unpack the drivers of this perceived responsibility to disclose even when transmission is deemed “impossible”. HIV exceptionalism is an important lens in understanding why some study participants who live with HIV may have felt compelled to disclose their status – particularly among a sample of people who have likely viewed and experienced HIV and HIV risk through an ‘exceptionalist’ lens, including during critical periods of their life course (e.g., HIV acquisition events; experiences living in a neighbourhood with very high HIV prevalence). Originally coined in 1991, HIV exceptionalism refers to the idea that HIV requires a response above and beyond “normal” public health interventions and should be treated differently in policy and law compared to other diseases, including those that are sexually transmitted and/or lethal [50, 51]. HIV exceptionalism continues to be reinforced by the criminalization of HIV non-disclosure and may partially explain participants’ perceived duty to disclose their seropositive status regardless of transmission risk. It is perhaps less likely that participants would have perceived the same level of duty to disclose other health conditions (e.g., Human Papillomavirus, Hepatitis C) to their sexual partners, illustrating the weight that HIV exceptionalism continues to carry in relation to other diseases, despite the immense progress that has been made with regard to medical treatments and prophylactics.

Similar to previous research a few of the authors of this article have conducted with other populations (e.g., young men) [14], participants in the current study tended to rationalize their support for the legal framework based on both misinformed assumptions and stigmatized beliefs about HIV that do not account for the current HIV intervention landscape. Unlike previous research undertaken by the authors, however, participants in the current study seemed less likely to emphasize the evolving intervention landscape (e.g., “U=U”) in their accounts, even after participants were asked about how undetectable/untransmittable viral loads ought to feature in considerations about the legal framework. As such, justifications of the highly criminalized legal framework tended to suggest that it would be effective at controlling HIV transmission, with little to no concern about effective treatment or prevention strategies. Furthermore, several participants advanced support for the framework based on media depictions of highly sensationalized accounts of HIV non-disclosure. Yet, several participants living with HIV also provided support for the framework based on their own personal experiences and the belief that, had the legal framework existed when they were infected with HIV in previous decades (i.e., prior to 1989), they would never have contracted the virus. Indeed, these arguments tended to assume that the overall effect of the legal framework would be effectively protective of public health, a claim that runs counter to the growing body of evidence that indicates that criminalization of HIV non-disclosure increases overall harm. As such, these findings underscore the extent to which the legal framework both reflects and contributes to widespread misunderstandings and stigmatized beliefs about HIV.

Hopefully, future changes to the HIV non-disclosure legal framework (e.g., provincial and federal prosecutorial guidelines, potential legislative reforms) will be advanced to both reflect the scientific consensus of HIV treatment and prevention [1], while also providing opportunities to address stigmatizing beliefs and misinformed assumptions about HIV. Fortunately, since empirical social scientific research was last conducted examining the perspectives of people navigating HIV risk in the context of the legal framework in their everyday lives [14], the Government of Canada has made new changes to directives and guidelines around prosecuting cases of HIV non-disclosure [19]. The new directives state that the federal government will no longer be prosecuting people living with HIV who have maintained a suppressed viral load. Though the new guidelines only apply to the Northwest Territories, Nunavut, and Yukon – territories in which judicial policy falls under federal jurisdiction – it is widely hoped that the provinces will follow suit with similar prosecutorial guidelines [19]. Recently, BC also made revisions to its own prosecutorial guidelines regarding non-disclosure, with the Crown Counsel being instructed to carefully balance the interests of the general public and the individual and sexual autonomy of victims while also ensuring that persons living with HIV are not subject to criminalization or stigmatization solely based on their illness [52]. While many advocates view these guidelines as an improvement from the current legal framework, many also believe it does not go far enough due to the fact that it does not clearly rule out the possibility of prosecuting those who have used a condom but do not have a suppressed viral load [53]. Nevertheless, it is hopeful that these new prosecutorial guidelines may have some effect in reducing HIV-related stigma, in addition to drawing attention to the ways individuals with HIV can achieve suppressed – and therefore untransmittable – viral loads.

Despite years of effective treatment therapies and educational efforts [54, 55], the findings underscore the extent to which HIV-related stigma continues to persist and undermine discourses of justice as well as public health objectives. Just as some of those living with HIV identified within their own narratives, it is extremely concerning that the criminalization of HIV non-disclosure is actively exacerbating stigma and perpetuating discriminatory attitudes and norms towards people living with HIV [23, 56] – including some of those that were identified in the current study. Nevertheless, HIV criminalization is only a partial explanation for the presence of HIV-related stigma in participant descriptions. The continued stigma towards people living with HIV suggests that the fear and anxieties produced through public discourse during the height of the HIV epidemic in the 1980s and 90s [57, 58] remains deeply entrenched in public consciousness and memory to this day. Further, since marginalized populations are overrepresented among those living with HIV, it is also reasonable to infer that HIV-related stigma serves partly as a proxy for societal attitudes towards these communities.

Despite positive changes to prosecutorial guidelines issued by the federal government, important questions around equity and justice still remain given that the HIV criminalization legal framework continues to apply to those with detectable viral loads. Baylis et al. argue that the greatest “burdens of pandemic” are often shouldered by the poorest and the most socially marginalized communities which, in itself, generates ethical issues [59]. The study population represents people who use drugs, a group among those disproportionately affected by HIV. The criminalization of drugs has reinforced the marginalization of people who use drugs, particularly those who are poor and racialized, while also discouraging them from accessing harm reduction and other health care services. Repressive anti-drug policies and practices have increased vulnerability to HIV among people who use drugs and discouraged their engagement in the HIV care continuum [36]. Studies continue to find that people living with HIV who use drugs are less likely to receive antiretroviral therapy [60]. Further, they are less likely to have suppressed viral loads while on treatment than other populations [61, 62]. By extension, while the criminalization of non-disclosure – in its ostensible attempt to protect ‘public health’ – has already been shown to be unfairly and selectively applied to poor, Indigenous, and Black communities [10, 20], it is highly concerning that any application of this framework will continue to disproportionately impact PWUD, thereby serving as a significant structural driver of inequity.

The three themes identified in this study point to the need for public health campaigns and programs to increase stigma reduction efforts and education about “U=U”. Reducing stigma could also entail correcting widely held assumptions about the disease by educating health care providers and the general public about the advances in biomedical technologies that have allowed many people living with HIV to achieve and sustain undetectable viral loads. Anti-stigma messages could also emphasize the significant role of socio-structural vulnerabilities that influence disease acquisition and transmission (e.g., poverty, housing insecurity, lack of access to harm reduction services, drug prohibition) instead of focusing on individual solutions and blame. Future public health campaigns and programs should be mindful of creating universal messaging that aims to reduce stigma for all people living with HIV to avoid reinforcement of stigma against those who have not achieved viral suppression.

To the knowledge of the authors, this is the first qualitative study to explore the perspectives of both people living with and without HIV who use illicit drugs on the criminalization of HIV non-disclosure in Canada. While the participant sample may be limited because it mostly represents the perspectives of people living within a small geographical area of the country and living in poverty, this study offers in-depth insights into the ways in which broader policies, structures, and norms shape attitudes and perceptions about the HIV legal framework among a population underrepresented in literature. Nevertheless, the participants in this study included a diverse group from other social categories such as gender, ethnicity, and sero-status. Despite this, this study is subject to several important limitations. The potential amplification of response biases (e.g., social desirability bias) may have also influenced the sorts of responses participants felt appropriate in the context of an interview. Previous research has shown that HIV-related stigma is often compounded by other mutually constituting stigmas based on sexuality, gender, race, class, drug use and employment in sex work – something authors were unable to further explore given limitations with the current study design. Future research should seek to explore these co-occurring stigmas with an intersectional lens in order to further identify complex barriers that hinder access to justice for people living with HIV. Furthermore, the scope of the findings may have been impacted by the sociodemographic features of the participant sample (e.g., age, sexual identity, and behaviour). While gay, bisexual, and men who have sex with men (MSM) account for a disproportionate number of HIV diagnoses in BC [32], they accounted for only a small percentage of those interviewed. Interviewing a higher number of people from these groups may have generated other insights regarding the HIV non-disclosure legal framework [10]. The range of perspectives presented by the data may have been limited by the number of participants (*n* = 60) that were invited to be part of the study. The study was designed to include 60 participants as it was believed this sample size would generate sufficient data and permit a diverse range of participants to be included in the study sample. However, the authors recognize that there are multiple perspectives on what constitutes data ‘saturation’ and that 60 participants may not have accounted fully for the variations at the intersections of ethno-racial identity, gender, class, sexual identity and so forth. Finally, future research in this area should include ‘natural experiments’ focused on the shifting frameworks in this area, including research involving people living with and without HIV and who live in regions affected by the federal government’s new prosecutorial guidelines.

## Conclusions

Understandings around HIV criminalization among participants in this study were significantly shaped by misguided understandings of how HIV is transmitted in the post-antiretroviral era, ongoing stigma towards people living with HIV, and personal experiences of contracting the disease or anecdotal ‘evidence’. While recent changes to federal prosecutorial guidelines surrounding the legal framework in Canada presents promising opportunities to address HIV stigma and the negative effects of criminalizing HIV non-disclosure, further reforms across the country’s judicial systems are needed to address the long-lasting effects on the underlying cultural and social conditions that cultivate discriminatory understandings of HIV risk and people living with HIV.

## Data Availability

The data in these findings are presented in this manuscript in support of themes as anonymized participant quotations. Due to the inability to sufficiently anonymize large qualitative datasets, further data cannot be made publicly available as per study approval by the research ethics board of Providence Health Care and the University of British Columbia.

## Abbreviations

HIV: Human immunodeficiency viruses
U=U: Undetectable viral loads are untransmittable
PWID: People who inject drugs
BC: British Columbia
VIDUS: Vancouver injection drug users study
ACCESS: AIDS care cohort to evaluate access to survival services
AIDS: Acquired immunodeficiency syndrome

## References

[1] Barre-Sinoussi F, Abdool Karim SS, Albert J, Bekker LG, Beyrer C, Cahn P (2018). Expert consensus statement on the science of HIV in the context of criminal law. J Int AIDS Soc.

[2] Picard A. Countries, including Canada, are prosecuting people with HIV because they misunderstand science, leading researchers say The Globe and Mail 2018 [cited 2019 May 31]. Available from: https://www.theglobeandmail.com/canada/article-countries-including-canada-are-prosecuting-people-with-hiv-because/.

[3] Network HJ. Unpublished data. 2018.

[4] Criminalization CCtRH. Developing a community consensus statement on ending unjust prosecutions for HIV non-disclosure. 2017.

[5] Smith J. Wilson-Raybould raises criminalization of HIV non-disclosure with counterparts The National Post 2017 [cited 2019 May 31]. Available from: https://nationalpost.com/pmn/news-pmn/canada-news-pmn/wilson-raybould-raises-criminalization-of-hiv-non-disclosure-with-counterparts.

[6] R. v. Cuerrier. Supreme Court of Canada; 1998.

[7] Mykhalovskiy E (2011). The problem of "significant risk": exploring the public health impact of criminalizing HIV non-disclosure. Soc Sci Med.

[8] Symington A. Criminalization confusion and concerns: The decade since the Cuerrier decision. Can HIV AIDS Policy Law Rev. 2009;14(1):5–10. 19606546

[9] Mykhalovskiy E, Betteridge G (2013). Who? What? Where? When? And with what consequences? An analysis of criminal cases of HIV non-disclosure in Canada. Can J Law Soc.

[10] HIV/AIDS. JUNPo. Ending overly broad criminalisation of HIV non-disclosure, exposure and transmission: critical scientific, medical and legal considerations. United Nations; 2013.

[11] R v. Mabior. Supreme Court of Canada; 2012.

[12] Loutfy MTM, Baril J-G, Montaner JSG, Kaul R, Hankins C (2014). Canadian consensus statement on HIV and its transmission in the context of criminal law. Can J Infect Dis Med Microbiol.

[13] Rodger AJ, Cambiano V, Bruun T, Vernazza P, Collins S, van Lunzen J (2016). Sexual activity without condoms and risk of HIV transmission in Serodifferent couples when the HIV-positive partner is using suppressive antiretroviral therapy. JAMA.

[14] Knight R, Krusi A, Carson A, Fast D, Shannon K, Shoveller J (2018). Criminalization of HIV non-disclosure: narratives from young men living in Vancouver, Canada. PLoS One.

[15] Siedner MJ, Triant V (2019). Undetectable = Untransmittable and your health: the personal benefits of early and continuous therapy for HIV infection. J Infect Dis.

[16] Cohen MS, Chen YQ, McCauley M, Gamble T, Hosseinipour MC, Kumarasamy N (2016). Antiretroviral therapy for the prevention of HIV-1 transmission. N Engl J Med.

[17] CATIE. Criminalization of HIV non-disclosure. Available from: https://www.catie.ca/en/hiv-canada/8/8-3#:~:text=However%2C%20the%20criminalization%20of%20non,sexual%20transmission%20of%20the%20virus. [cited 2019 May 31].

[18] Canadian HIV/AIDS Legal Network. The Criminalization of HIV Non-Disclosure in Canada: Current status and the need for change. 2019. Available from: http://www.aidslaw.ca/site/the-criminalization-of-hiv-non-disclosure-in-canada-report/?lang=en. [cited 31 May 2019].

[19] Brown D. Justice Department issues new guidelines on prosecution for non-disclosure of HIV status CBC News 2018 [cited 2018 May 31]. Available from: https://www.cbc.ca/news/canada/toronto/canada-prosecutions-hiv-non-closures-cases-1.4929292.

[20] Shimeless H, Bailey R (2011). African and Caribbean Council on HIV/AIDS in Ontario.

[21] Wilson C. The impact of the criminalization of HIV non-disclosure on the health and human rights of “black” communities. Health Tomorrow: Interdisciplinarity Internationality. 2013;1(1):109–143.

[22] O'Byrne P, Bryan A, Woodyatt C (2013). Nondisclosure prosecutions and HIV prevention: results from an Ottawa-based gay men's sex survey. J Assoc Nurses AIDS Care.

[23] Arreola S, Santos G-M, Beck J, Sundararaj M, Wilson PA, Hebert P (2014). Sexual stigma, Criminalization, investment, and access to HIV services among men who have sex with men worldwide. AIDS Behav.

[24] Adam BD, Elliott R, Corriveau P, English K (2013). Impacts of Criminalization on the everyday lives of people living with HIV in Canada. Sex Res Soc Policy.

[25] Harsono DGC, O’Keefe E, Lazzarini Z. Criminalization of HIV Exposure: A Review of Empirical Studies in the United States. AIDS Behavior. 2017;21(1):27–50. 10.1007/s10461-016-1540-5 PMC5218970 27605364

[26] Mykhalovskiy E BJ, Sanders C, Jones M. The public health implications of criminalizing HIV non-disclosure, exposure and transmission: Report of an international workshop.; 2014.

[27] Patterson S, Kaida A, Ogilvie G, Hogg R, Nicholson V, Dobrer S (2017). Awareness and understanding of HIV non-disclosure case law among people living with HIV who use illicit drugs in a Canadian setting. Int J Drug Policy.

[28] Krüsi A, Czyzewski K, Magagula P. Through our eyes. Medicine Anthropology Theory | An open-access journal in the anthropology of health, illness, and medicine. 2017;4(3).

[29] Krusi A, Ranville F, Gurney L, Lyons T, Shoveller J, Shannon K (2018). Positive sexuality: HIV disclosure, gender, violence and the law-a qualitative study. PLoS One.

[30] World Health Organization. People Who Inject Drugs. Available from: https://www.who.int/hiv/topics/idu/about/en/. [cited 2019 May 31].

[31] AVERT. People who inject drugs, HIV and AIDS [updated October 10, 2019.

[32] Control. BCfD. HIV in British Columbia: Annual Surveillance Report 2016. 2018.

[33] Neale J, Tompkins C, Sheard L (2008). Barriers to accessing generic health and social care services: a qualitative study of injecting drug users. Health Soc Care Community.

[34] Lang K, Neil J, Wright J, Dell CA, Berenbaum S, El-Aneed A. Qualitative investigation of barriers to accessing care by people who inject drugs in Saskatoon, Canada: perspectives of service providers. Subst Abuse Treat Prev Policy. 2013;8(35):1–11. 10.1186/1747-597X-8-35 PMC3850796 24079946

[35] Matsuzaki M, Vu QM, Gwadz M, Delaney JAC, Kuo I, Trejo MEP (2018). Perceived access and barriers to care among illicit drug users and hazardous drinkers: findings from the seek, test, treat, and retain data harmonization initiative (STTR). BMC Public Health.

[36] UNAids. Prevention Gap Report. 2016.

[37] Canada S. Census of population. 2016.

[38] Olding M, Enns B, Panagiotoglou D, Shoveller J, Harrigan PR, Barrios R (2017). A historical review of HIV prevention and care initiatives in British Columbia, Canada: 1996-2015. J Int AIDS Soc.

[39] Control BCfD. HIV in British Columbia: annual surveillance report 2015. 2017.

[40] Bramham D. More needed to redress the tragic fact that Indigenous people are disproportionately victims of opioid crisis Vancouver Sun. 2019. Available from: https://vancouversun.com/opinion/columnists/daphne-bramham-more-needed-to-redress-the-tragic-fact-that-indigenous-people-are-disproportionately-victims-of-opioid-crisis. [cited 2019 May 1].

[41] Palinkas LA, Horwitz SM, Green CA, Wisdom JP, Duan N, Hoagwood K (2015). Purposeful sampling for qualitative data collection and analysis in mixed method implementation research. Admin Pol Ment Health.

[42] Creswell J, Klassen AC, Plano Clark VL, Smith KC. Best practices for mixed methods research in health sciences. Office Behav Soc Sci Res. 2011;2011:1–39.

[43] Patton (2002). Designing qualitative studies. Qualitative research and evaluation methods.

[44] Constructing CK, Theory G (2014). 2nd edition ed.

[45] Boeije H (2002). A purposeful approach to the constant comparative method in the analysis of qualitative interviews. Qual Quant.

[46] Hallberg LRM (2009). The “core category” of grounded theory: making constant comparisons. Int J Qual Stud Health Well Being.

[47] Kapiri L, Norheim OF, Martin DK (2007). Priority setting at the micro-, meso, and macro- levels in Canada, Norway and Uganda. Health Policy.

[48] Braun V, Clarke V (2006). Using thematic analysis in psychology. Qual Res Psychol.

[49] Clarke V, Braun V (2016). Thematic analysis. J Posit Psychol.

[50] Oppenheimer GM, Bayer R (2009). The rise and fall of AIDS exceptionalism. Virtual Mentor.

[51] Smith JH, Whiteside A (2010). The history of AIDS exceptionalism. J Int AIDS Soc.

[52] Crown Counsel Policy Manual, (2019).

[53] New Policy for BC Prosecutors Still Harms People Living with HIV [press release]. April 23, 2019.

[54] Taylor D. In Your Face: AIDS posters confront stigma CATIE. 2014.

[55] Davis J. Evolution of an epidemic: 25 years of HIV/AIDS media campaigns in the US. The Henry J Kaiser Family Foundation; 2006 June 2006.

[56] Parker R, Aggleton P (2003). HIV and AIDS-related stigma and discrimination: a conceptual framework and implications for action. Soc Sci Med.

[57] Persson A, Newman C (2008). Making monsters: heterosexuality, crime and race in recent Western media coverage of HIV. Sociol Health Illn.

[58] Squire C, Ussher J (1997). Body talk: the material and discursive regulation of sexuality, madness and reproduction. AIDS Panic.

[59] Baylis F, Kenny NP, Sherwin S (2008). A relational account of public health ethics. Public Health Ethics.

[60] Westergaard RP, Ambrose BK, Mehta SH, Kirk GD. Provider and clinic-level correlates of deferring antiretroviral therapy for people who inject drugs: a survey of North American HIV providers. J Int AIDS Soc. 2012;15(1):10. 10.1186/1758-2652-15-10 PMC3306203 22360788

[61] Lourenco L, Colley G, Nosyk B, Shopin D, Montaner JS, Lima VD (2014). High levels of heterogeneity in the HIV cascade of care across different population subgroups in British Columbia, Canada. PLoS One..

[62] Martin L, Houston S, Yasui Y, Wild TC, Saunders LD. Rates of initial virological suppression and subsequent virological failure after initiating highly active antiretroviral therapy: the impact of aboriginal ethnicity and injection drug use. Curr HIV Res. 2010;8(8):649–658. 10.2174/157016210794088227 PMC4428381 21187007

